# Patient and Provider Perspectives About the Use of Patient-Generated Health Data During Pregnancy: Qualitative Exploratory Study

**DOI:** 10.2196/52397

**Published:** 2024-05-08

**Authors:** Sarah R MacEwan, Ramona G Olvera, Pallavi Jonnalagadda, Naleef Fareed, Ann Scheck McAlearney

**Affiliations:** 1 Division of General Internal Medicine College of Medicine The Ohio State University Columbus, OH United States; 2 Center for the Advancement of Team Science, Analytics, and Systems Thinking in Health Services and Implementation Science Research College of Medicine The Ohio State University Columbus, OH United States; 3 Department of Biomedical Informatics College of Medicine The Ohio State University Columbus, OH United States; 4 Department of Family and Community Medicine College of Medicine The Ohio State University Columbus, OH United States

**Keywords:** patient-generated health data, patient-centered care, obstetrics, postpartum period, qualitative methods

## Abstract

**Background:**

There is increasing interest in using patient-generated health data (PGHD) to improve patient-centered care during pregnancy. However, little research has examined the perspectives of patients and providers as they report, collect, and use PGHD to inform obstetric care.

**Objective:**

This study aims to explore the perspectives of patients and providers about the use of PGHD during pregnancy, including the benefits and challenges of reporting, collecting, and using these data, as well as considerations for expanding the use of PGHD to improve obstetric care.

**Methods:**

We conducted one-on-one interviews with 30 pregnant or postpartum patients and 14 health care providers from 2 obstetrics clinics associated with an academic medical center. Semistructured interview guides included questions for patients about their experience and preferences for sharing PGHD and questions for providers about current processes for collecting PGHD, opportunities to improve or expand the collection of PGHD, and challenges faced when collecting and using this information. Interviews were conducted by phone or videoconference and were audio recorded, transcribed verbatim, and deidentified. Interview transcripts were analyzed deductively and inductively to characterize and explore themes in the data.

**Results:**

Patients and providers described how PGHD, including physiologic measurements and experience of symptoms, were currently collected during and between in-person clinic visits for obstetric care. Both patients and providers reported positive perceptions about the collection and use of PGHD during pregnancy. Reported benefits of collecting PGHD included the potential to use data to directly inform patient care (eg, identify issues and adjust medication) and to encourage ongoing patient involvement in their care (eg, increase patient attention to their health). Patients and providers had suggestions for expanding the collection and use of PGHD during pregnancy, and providers also shared considerations about strategies that could be used to expand PGHD collection and use. These strategies included considering the roles of both patients and providers in reporting and interpreting PGHD. Providers also noted the need to consider the unintended consequences of using PGHD that should be anticipated and addressed.

**Conclusions:**

Acknowledging the challenges, suggestions, and considerations voiced by patients and providers can inform the development and implementation of strategies to effectively collect and use PGHD to support patient-centered care during pregnancy.

## Introduction

Patient-centered care can improve health outcomes by addressing each patient’s individual needs, increasing patients’ ownership of their health decisions, and improving patient-physician relationships [1-4]. In obstetrics, patient-centered care is recognized as important, yet providing this care during pregnancy remains challenging [5-8] as providers need to gather information from patients about their health and well-being throughout their pregnancies. To facilitate the gathering of person-centered information, there is increasing interest in the role of patients in reporting health-related data to their providers to inform patient-centered care.

Patient-generated health data (PGHD) is any health-related information that is gathered from or recorded directly by patients [9-11]. These data include self-reported symptoms, health behaviors, and physiologic measurements. While PGHD is frequently gathered during in-person appointments, opportunities also exist to collect these data between visits. To date, the collection of PGHD between in-person appointments to inform patient care has largely focused on physiologic measurement data, such as blood glucose levels and blood pressure [12-18]. The collection of these measurements is of particular interest during pregnancy, as diabetes and hypertension present common and serious risks to both mothers and their babies [19-27]. Although other aspects of patient-reported health status have been identified as important to informing patient care during pregnancy, such as quality of life, pain, and mental health status [28], limited evidence exists about the collection of these data between in-person appointments, or about the use of this information to inform patient care.

Patient sharing of PGHD between in-person appointments has been shown to improve patient-provider communication [29], enhance patients’ involvement in their clinical visits [30], and increase insight into patients’ health between clinical visits [31]. While evidence is building to understand if and how the use of PGHD can improve health outcomes [32], it is largely understudied in the context of pregnancy [33,34]. The use of PGHD may be particularly beneficial to improve obstetric care, as pregnancy encompasses a period during which many individuals experience frequent changes in their health status, and there are potentially serious complications that can occur that can impact maternal and infant health outcomes.

While there is growing interest in implementing strategies to collect PGHD, there has been little exploration of the experiences of patients and providers as they share and receive these data [35]. When implementing strategies to collect and use PGHD to inform patient care, technological considerations, such as integration of data into the electronic health record [12-14], have been at the forefront of research efforts. Less attention has been paid to the experiences of patients and providers as they collect and report PGHD, their perceptions of the impact of using PGHD, and the practical considerations that are critical to the implementation of strategies to expand the collection and use of these data. Exploring the perspectives of both patients and providers is, therefore, critical to improving our understanding of their needs and preferences, many of which should be acknowledged and addressed in clinical guidelines, reimbursement strategies, policy, and rules of engagement for the collection and use of PGHD [35].

This study aims to explore the perspectives of pregnant patients about sharing PGHD and the perspectives of obstetric providers about collecting and using PGHD for clinical decision-making during pregnancy. We were interested in understanding the benefits of using PGHD, as perceived by patients and providers, as well as the challenges that each group noted. Finally, we sought to gather patients’ and providers’ suggestions for ways to improve or expand the collection and use of PGHD during pregnancy.

## Methods

### Study Design and Setting

This exploratory qualitative study was guided by a constructivist and interpretivist research paradigm [36]. Through one-on-one interviews, we sought to explore the perspectives of our study participants within the unique contexts of their roles and experiences. We recognize the reflexivity of our research team in our data collection and the interpretation of our study findings, where research team members included bachelor-trained and doctoral-trained health services researchers. The reporting of our findings is guided by the Standards for Reporting Qualitative Research checklist [37].

We conducted one-on-one interviews with patients and providers from 2 obstetrics and gynecology ambulatory care clinics at The Ohio State University Wexner Medical Center, an academic medical center (AMC), between September 2020 and January 2021. Patients who were 18 years or older, pregnant or up to 90 days postpartum, and spoke English were eligible to participate. Eligible providers included physicians and nurses who worked in either or both clinics. Purposeful sampling was used to recruit patients to the study by telephone call inviting their participation, while providers were recruited by email. Emails sent to providers included a Qualtrics survey link to indicate their interest in participating and their preference for a telephone or videoconference interview.

### Data Collection

Separate semistructured interview guides were used to support data collection from patients and providers (Multimedia Appendices 1 and 2). These interview guides were developed by 3 members of the research team (ASM, SRM, and NF) who are doctoral-trained health services researchers. Questions for patients asked about their experiences and preferences for sharing information with their providers, including self-reported symptoms and physiologic measures. The patient interview guide was tested with 2 individuals—1 pregnant and 1 post partum—before finalizing the interview questions. Provider interviews asked about current strategies for collecting patient-generated information, opportunities to implement new strategies to collect this information in the future, and potential challenges faced when collecting this information.

Patient interviews were conducted by 2 female members of the research team who were either doctoral-trained or bachelor-trained (SRM and Abigail Petrecca). Patients did not know their interviewer prior to their interview. Patients were contacted by telephone and were given the option to complete the interview at the time of the call or schedule their interview at another time that was convenient for them.

Providers who were interested in participating in the study from the initial recruitment email provided a preferred time to schedule either a phone or videoconference (ie, Zoom) interview. Provider interviews were completed by 2 members of the research team, 1 female and 1 male, who were both doctoral trained (SRM and NF). Most providers did not know their interviewer prior to their interview; however, some providers had met their interviewer in a professional capacity prior to their interview. All interviews were audio-recorded and transcribed verbatim to permit rigorous qualitative analysis.

### Data Analysis

We used thematic analysis to code the transcripts from all interviews [38]. First, we created separate preliminary coding dictionaries for the patient and provider interview transcripts based on questions from the semistructured interview guides (Multimedia Appendix 3). Three doctoral-trained or bachelor-trained members of the research team (SRM, NF, and Holly Heffer) then coded 2 patient transcripts and 2 provider transcripts using these preliminary coding dictionaries. The coders met to discuss discrepancies in initial coding and refined the coding dictionary. Two of the initial coders (SRM and Holly Heffer) individually coded the remaining transcripts using the refined coding dictionary, meeting frequently to ensure consistency between the coders. After this deductive coding, the 2 coders met to discuss emergent subthemes they identified during the coding process and inductively developed subcodes that were added to the coding dictionary. One coder (Holly Heffer) then applied these subcodes to all the transcripts. The coding team reached thematic saturation based on consensus among coders that themes fully covered emergent topics across transcripts [39]. ATLAS.ti software (ATLAS.ti Scientific Software Development GmbH) was used to support the coding and analysis process.

### Ethical Considerations

The Ohio State University’s institutional review board approved this study (2020B0038). Participation was voluntary and all participants provided verbal informed consent. Interview transcripts were deidentified to protect participant privacy and confidentiality. Patients and nonphysician participants received a US $25 gift card in appreciation for their participation.

## Results

### Participant Characteristics

Interviews were conducted with 30 patients and 14 providers. Patients had a mean age of 30 years and included 22 pregnant individuals (mean gestational age: 28.6 weeks) and 8 postpartum individuals (mean weeks postpartum: 6.3). Providers included 9 physicians and 5 nurses. Patient interviews lasted an average of 16 minutes, while provider interviews lasted an average of 26 minutes.

### Thematic Analysis

#### Overview

Three themes were identified across patient and provider interviews: (1) current collection of PGHD during pregnancy, (2) suggestions for reporting PGHD during pregnancy, and (3) the impact of reporting PGHD during pregnancy. A fourth theme was identified only from provider comments: (4) considerations for expanding the collection of PGHD during pregnancy. Themes and their respective subthemes are discussed below and summarized in Table 1.

**Table 1:** Summary of themes and subthemes.

Themes and subthemes			Description
**Current collection of PGHD^a^ during pregnancy, by health concern and measurement**			
	**Diabetes**		
		Blood glucose	4-8 daily blood glucose values reported weekly through email
		Symptoms (eg, indications of ketoacidosis)	Only reported at in-person appointments
	**Hypertension**		
		Blood pressure	Out-of-range values reported by phone
		Symptoms (eg, indications of pre-eclampsia)	Only reported at in-person appointments
	**Mental health**		
		Perceptions of mood, anxiety, and depression	Only reported at in-person appointments
	**Fetal movement**		
		Kick counts	Decreases in movement reported by phone
	**Other symptoms of pregnancy complications**		
		Nausea, bleeding, cramping, discharge, vaginal pressure, dizziness, fainting, itching, and pain	Worsening symptoms reported by phone or patient portal messaging
**Suggestions for reporting PGHD during pregnancy**			
	Use patient portal for PGHD collection		Reporting PGHD in the patient portal could allow data to be stored in a way that is accessible to both patients and all members of the health care team.
	Use reminders to collect PGHD		Reminders to take and record measurements could increase adherence with PGHD reporting.
	Collect information about symptoms		Collecting symptoms, in addition to physiologic measurements, could facilitate the identification of concerns or complications.
	Expand collection of PGHD to health concerns that are currently only assessed in person		Collection between in-person appointments could improve clinical awareness of information related to fetal movement and mental health concerns.
**Impact of reporting PGHD during pregnancy between in-person appointments**			
	Informs patient care		Collecting PGHD facilitates the identification of concerns and the adjustment of patient care.
	Encourages patient involvement in their care		Reporting PGHD increases attention to and awareness of health.
**Considerations for expanding the collection of PGHD during pregnancy between in-person appointments**			
	Considerations for the patient role		Target and tailor PGHD collection for each patient’s clinical needs. Provide patient guidance for reporting information that may be difficult to quantify (eg, nausea, severity of bleeding).
	Considerations for the provider role		Present data in a way that conveys necessary information and trends over time (eg, alert to abnormal values that suggest clinical concern). Assign roles and responsibilities for who is reviewing and responding to PGHD as it is collected.
	Considerations for unintended consequences		Have mechanisms in place to identify and escalate emergencies. Recognize potential increase in nonemergent concerns and subsequent clinical burden and cost.

^a^PGHD: patient-generated health data.

#### Current Collection of PGHD During Pregnancy

Patients and providers described the current collection, reporting, and use of PGHD during pregnancy. Patients and providers reported that the majority of PGHD were collected or reported during in-person appointments. It was noted to be less common for patients to report PGHD or for providers to collect PGHD between their appointments.

Providers described collecting PGHD related to symptoms, as well as self-reported physiologic measurements including blood pressure and blood glucose values, and noted using these data to direct patient care. Although information about symptoms was predominantly collected in person, providers reported informing patients to call the AMC if they experienced any concerning or worsening symptoms between visits. For example, 1 provider described:

I tell patients to keep a log [of fetal kicks] if they feel like, you know, to get a basis for what their babies do, because some babies are more active and some are less active. So, typically we're not like reviewing those logs. I just tell them to keep that information so if they noticed that it's less frequent, they can, you know, let us know.

provider 3

Providers commented that they asked some patients to regularly measure their blood pressure at home between appointments and contact their provider if their blood pressure exceeded a certain limit. Providers also reported that they asked patients with diabetes to track their blood sugar levels at home (taking between 4 and 8 daily measurements) and share those values with them each week. Patients most frequently reported sharing blood sugar values with a clinic nurse via email.

#### Suggestions for Reporting PGHD During Pregnancy

Patients and providers described several opportunities to improve or expand the collection of PGHD between in-person appointments.

##### Use Patient Portal for PGHD Collection

For physiologic measurements that were already being reported between appointments to monitor hypertension and diabetes (ie, self-reported blood pressure and blood glucose measurements), patients suggested that it might be better to report this information in the AMC’s patient portal, where the data could be stored and available for the whole care team to view. One patient suggested:

I think I'd rather have it in the patient portal because then it's like recorded. It's logged, everyone has access to it. So, it's not just one person has an email, you know, the whole staff team could have availability of it and then you have it.

patient 13

##### Use Reminders to Collect PGHD

Patients and providers also commented that reminders might improve adherence to taking and reporting these measurements. As 1 provider suggested:

We probably could be better about being sure that patients are potentially even sent reminders about like how often they should be checking their blood pressures ...whether it be morning and night or just one time during the day. You know, it's sending a reminder to them to be like, “Hey, can you enter your blood pressure for the day?”

provider 9

##### Collect Information About Symptoms

For patients with diabetes or hypertension, providers suggested collecting additional information about symptoms between in-person appointments; for example, asking about complications among patients with diabetes, as 1 provider suggested: “With the diabetic patients, I think it would be helpful to know, are you having anything that could be signs and symptoms of ketoacidosis” (provider 5). Another provider suggested asking about symptoms of pre-eclampsia: “I think certainly if we have a patient who has preeclampsia, asking them for symptoms like headache, change in vision, things like that would be important” (provider 8).

##### Expand Collection of PGHD to Health Concerns That Are Currently Only Assessed in Person

Patients and providers also proposed that it could be useful to collect PGHD between appointments related to health concerns that were currently only assessed in person, including changes in fetal movement and evaluating mental health status. One provider described the opportunities to collect information about fetal movement:

With like you mentioned earlier, fetal movement, there’s no formalized way that we do that except, you know, talk to people in office visits. But if you want to collect data, that would be a good thing to do if patients could just weekly send them something saying, yes fetal movement has been the same, or fetal movement has been less, or things like that.

provider 5

With regard to evaluating mental health status, 1 patient recommended:

Maybe the postpartum depression, stuff like that, maybe have them look more into that. Because the only time I've done the questionnaire for that was when I took my twins to the pediatrician.

patient 11

A provider echoed this suggestion:

I think if there could be some sort of system where it tracks people's mood and then if it was really bad, you know some certain threshold, that it sent a notification to the provider. I think that would be good, especially for postpartum depression.

provider 2

#### Impact of Reporting PGHD During Pregnancy Between In-Person Appointments

Patients and providers also noted the positive impacts of reporting PGHD during pregnancy, including that sharing PGHD helped to inform patient care and helped to encourage patients’ involvement in their care.

##### Informs Patient Care

First, providers commented that reporting PGHD allowed them to identify issues and adjust patient care between clinical visits. For example, collecting blood sugar values allowed providers to adjust diabetes medication, as 1 patient with pregestational diabetes described, “It [reporting blood sugar values] helped, it definitely helped regulate. It helped them to figure out how to adjust my [insulin] pump to regulate my diabetes in general” (patient 23). One provider described how having information between in-person visits helped them make health care decisions for their patients:

I think that they empower you to feel like you're making the right decision and care for the patient. I mean, I know when I have, when I have the information documented and I have it to work off of, I think two ways in which I think it’s helpful: one, it gives me the comfort level as the provider that I'm making an informed decision about their care. I think, in particular, about the diabetic patients and the hypertensive patients, it's good to have that information so that you know that you can make changes to their care that feel appropriate and safe.

provider 3

Providers also noted that knowing PGHD between in-person appointments could help them adjust patient care in a timely manner:

The blood pressure, blood sugars, you know, in between visits, if we knew that we were having issues, you know, we would be able to assist them quicker than you know, waiting until the next appointment. Or even with postpartum depression, or, you know, fetal movement counts. You know, those things we would be able to address quicker if we did it in between visits. If we were able to track it in between visits.

provider 15

##### Encourages Patient Involvement in Their Care

Patients and providers also reflected that reporting PGHD could encourage patients’ involvement in their care. One patient explained their increased attention to their health when asked to report information between in-person appointments:

I just feel like you're more aware of things and pay more attention to things if you are like sending that information in between appointments...sometimes you wait till you almost have your appointment and then you start paying attention to things. Where if you're doing it like every week or throughout, then you’re just more aware, and you're basically paying more attention to what's going on.

patient 28

Another patient described their increased sense of control in their health care with regard to reporting their blood glucose levels: “With what I have done with the insulin and some concerns with the gestational diabetes at the beginning, it does make me feel more in control, and it does make me feel like I have more say in my care” (patient 29). A provider’s comment echoed this perspective: “I do think it will give patients more ownership of their care” (provider 5).

Providers also expressed that collecting PGHD could help them understand patients better and potentially strengthen the patient-provider relationship, which might similarly encourage patients’ involvement in their care: “I think it would just allow for a more like solid or strengthened patient-doctor relationship that you know, hopefully the patient would feel that I'm more involved and checking in on her, and kind of aware of what's going on in between visits” (provider 4).

#### Considerations for Expanding the Collection of PGHD During Pregnancy Between In-Person Appointments

Finally, providers mentioned several considerations when discussing opportunities to expand the collection of PGHD to support obstetric care. These fell into the following categories: considerations for the patient role, considerations for the provider role, and considerations for unintended consequences.

##### Considerations for the Patient Role

With respect to patients’ roles in reporting PGHD, providers noted the importance of targeting and tailoring instruments (eg, questionnaires) to collect this information. Providers stressed that PGHD collection from each patient should be personalized to that patient’s individual clinical needs. As 1 provider described:

Linking certain diagnoses to it and certain conditions to it, it makes them much more targeted and tailored approaches rather than just kind of a “here’s everything you could possibly answer.” And that would be completely overwhelming to people and completely turn them off.

provider 9

Providers also commented about the need for guidance when asking patients to report information that may be hard to quantify, including the perceived severity of symptoms that are subjective. One provider described this challenge in the context of nausea:

I think nausea is hard because I think so many women also experience nausea. And we try to manage those women as an outpatient as best as we can. And I think sometimes it's patient perspective that helps. You know, you can have someone who's really nauseous and vomits all the time and they're able to manage that at home with medications. ...And then you have other women who vomit but they feel like their amount of nausea is something they need to be seen for. Their amount of nausea and their amount of vomiting objectively may be less than someone else who's controlling symptoms at home. So, I think that would be a little tricky.

provider 2

In another example, a provider explained this consideration in the context of bleeding:

You know, bleeding is a little bit harder because that's a little bit, so subjective. But it can be, I think there's ways to potentially quantify that to people's satisfaction, such that they would feel okay with it. Like in the sense of you know, if you have spotting on your tissue, okay, you know, did you see it again? Or like, check again with like another wipe or in like another like two hours, if it's still there come in. Or if it's fully filling the tissue paper or even like a panty liner come in, those types of things.

provider 9

##### Considerations for the Provider Role

With regard to the provider’s role in reviewing and responding to PGHD, providers stressed the importance of how the data would need to be presented to them. Providers reflected that, depending on the type of PGHD, they may not need to review every value or data point, but rather the data should be presented in a way to alert them to abnormal values that would be cause for concern. One provider gave the example:

I mean some things we need to know, like blood sugars. We have to know every single blood sugar. But you know, perhaps something like a fetal movement count, we don't really need to know about that until it's abnormal.

provider 12

Providers also commented on the importance of presenting the data in a way that could help them understand trends over time (eg, tables or charts), rather than going through data presented as text alone: “If we have a formalized portal, that would be great, that would come up with a you know, a graph or a table rather than have to wade through a bunch of texts” (provider 5).

Additionally, providers identified the importance of assigning roles and responsibilities among the care team to ensure that PGHD was collected appropriately, promptly reviewed, and acted upon by the care team. One provider explained these considerations:

I’d say there's the systemic issue of you know, who is responsible for making sure that information is read? When is it supposed to be read? And what ways are we supposed to respond to it? Who's supposed to be responding to it? How frequently are they supposed to be checking?

provider 13

Considering roles and responsibilities was critical due to the additional workload these processes presented to the health care team, as 1 provider commented:

During pregnancy, my concern with collecting all the information would be about who is assessing it. So, if we are sending out text messages once a week or every day, making sure that there's a nurse who's going to be really thoughtful about looking at it so that the concerns, symptoms, complaints are not automatically pushed to the physician to review and look at. I think there's already a lot of work being done and physician burnout, and so my concern would be that with all this extra data, is that overwhelming the physician’s already limited time and brain capacity during the day to kind of review all that information?

provider 8

##### Considerations for Unintended Consequences

Finally, providers described considerations regarding the potential unintended consequences of collecting PGHD. First, providers noted the need to identify and react to emergencies as indicated by patients’ responses. One provider gave an example in the context of a patient reporting changes in fetal movement:

If there was a way for the system to automatically tell the patient if she said, “I'm not feeling the baby move, I have decreased fetal movement,” then there should be no delay. It should be like an automatic response to come to the hospital and get evaluated. That is the only thing. I feel like there’s some liability there if a rare event or something would happen.

provider 4

Another provider described how processes might be designed to help identify emergencies in the context of identifying pre-eclampsia:

We have the potential for automation to kind of play a factor in the sense that you know, if you have certain triggering blood pressure thresholds that would, you know, rapidly bring that to the attention of somebody, or more specifically guide a patient through, okay you have this blood pressure, is this a second blood pressure you've already checked because it was elevated before? If not, you know, re-check it in 15 minutes after you've sat down, relaxed, all that thing. Or if this blood pressure is still severely elevated above this level after 15 minutes, you know, please, you know, report to the like emergency room or, you know, call this number specifically so we can get you in touch with the provider and guide you through the care process.

provider 9

In addition to identifying and reacting to emergencies, providers also expressed consideration that the collection of PGHD may increase the number of nonemergent concerns being reported during pregnancy. One provider explained their consideration:

I think my biggest concern would just be with, with extra data comes up with extra medical intervention. That would be my biggest concern. I think them reporting it would be fine. But would we be more apt to bring people into the hospital then, to assess these symptoms? More testing? When ultimately maybe they were, you know, most of them were fine.

provider 8

Some providers questioned whether reporting PGHD would result in patients reporting a greater number of concerns, which could also potentially increase medical costs and cause extra work for clinic staff:

I'd be a little bit worried that we would get a lot of responses that we would be following up on that wouldn't necessarily amount to anything. And that would be a lot of extra work for the staff in bringing those patients in and tracking those outcomes on so many patients that we have.

provider 2

## Discussion

### Principal Results

Our qualitative analysis provides insight into patients’ and providers’ perspectives on the value and challenges of collecting and using PGHD to inform obstetric patient care. While the use of PGHD during pregnancy has most commonly been reported for objective physiologic measurements (eg, blood glucose and blood pressure) [40,41], our patient and provider participants also expressed interest in the collection of subjective measurements including anxiety, depression, and symptoms indicative of pregnancy complications. Recognizing the interest in PGHD beyond physiologic measurements is important, as studies have demonstrated that the collection of a range of different types of PGHD can improve understanding of the unique context of each patient’s pregnancy. For example, pregnant women with heart disease identified general well-being, mental health, fatigue, and quality of life as important topics for which PGHD, in addition to clinical outcomes specific to cardiac function, may be critical information to share with their providers [42]. Similarly, in a study of pregnant women with gestational diabetes, the collection of PGHD including patient perspectives of anxiety, self-knowledge about diabetes, and social support has been proposed to provide a more comprehensive picture of the patient that helps explain why some patients experience challenges with blood glucose management [43]. Increasing holistic awareness of the patient through the collection of PGHD may also help providers target interventions (eg, education) as a means to improve maternal health outcomes [44].

The growing emphasis on patient-centered care during pregnancy has brought to light the potential of PGHD as an important factor that can enhance the delivery of value-based care [23,28,45-47]. Evidence is growing surrounding the use and effectiveness of strategies for using PGHD to inform obstetric care. For example, uploading self-reported measures of blood glucose to a patient portal has been associated with improvements in glycemic control in a sample of pregnant and nonpregnant patients [13], and continuous monitoring of blood glucose has been associated with improved neonatal outcomes [46,48]. Evidence will continue to grow from several ongoing clinical trials on this topic, with the greatest focus of these studies on patient-generated physiologic measurements [49,50].

Despite interest in expanding the use of PGHD during pregnancy, little work has investigated the perspectives of stakeholders in the use of PGHD, including those of pregnant individuals providing this information and the providers receiving and using this information to improve obstetric care. While patients and providers in our study commented about the clinical benefits of using PGHD, for which there is a mixture of evidence in the literature [15,51,52], they also suggested the additional benefit of improving patients’ involvement in their care through the collection of PGHD. In nonpregnant populations, the collection of PGHD has been recognized to create an opportunity for self-reflection among patients that can encourage patient-provider communication, which, in turn, increases provider awareness of patient concerns [53]. Similarly, others have suggested additional benefits of using PGHD beyond their impact on health outcomes, such as improved patient satisfaction with care as a result of improved patient-provider communication [29,45,54].

In imagining the expanded use of PGHD to inform obstetric care, providers in our study explained several considerations in the practical implementation of strategies to collect and use this information. These considerations included questions surrounding the patient’s role in reporting PGHD, the provider’s role in using PGHD, and the potential unintended consequences of collecting PGHD. These considerations bring to light how challenging it can be to introduce PGHD into clinical practice. Furthermore, it will be important to evaluate these considerations within the unique context of the multiple ways in which PGHD can be collected, synthesized, and displayed. Examples of methods in operation or development for these purposes include the use of electronic health records and patient portals [12], automated SMS text messages [55-57], mobile health apps [29], wearable devices [58,59], and remote patient monitoring systems [60,61].

While interest in expanding the use of PGHD during pregnancy was expressed by providers in our study, there is little guidance on the collection of PGHD and a paucity of research on using PGHD in this patient population [33,34]. Furthermore, the infrastructure to measure and synthesize PGHD is not extensive in the United States [45]. For example, challenges noted in the literature include how PGHD can be collected, how to best interpret PGHD, and how meaningful thresholds of PGHD are indicative of an action required by the patient or provider [62-64]. Notably, many of the approaches to address these challenges will be specific to providers, their health care systems, and the patient populations they serve.

One mechanism to increase our collective knowledge surrounding the collection and use of PGHD in pregnancy is through the existing call to action for randomized controlled trials in maternal health to standardize the selection, collection, and reporting of outcomes based on PGHD that reflect the perspectives of study participants [65]. Such an endeavor could facilitate the translation of research into clinical practice for PGHD collection and use while increasing contextual knowledge about patient health and health care experiences that can be used to improve the patient-centeredness of obstetric care. Future research should focus on the evaluation of methods for using PGHD in obstetric care to begin to build evidence and inform implementation strategies for this practice.

### Limitations

One limitation of our study is that the participant population was drawn from a single AMC. Our findings, therefore, represent perspectives specific to the patient and provider populations at our clinical sites. In addition, while our study population included both patients and providers, our analytical approach was not designed to identify convergence or divergence of perspectives across groups. Future research that explicitly assesses similarities and differences in the perspectives of patients and providers on these topics is important to inform interventions for PGHD collection and reporting that meet the needs and preferences of both stakeholder groups. We conducted this study during the COVID-19 pandemic, a period during which pregnant individuals may have experienced changes in their clinical care due to the many impacts of the pandemic. Due to restrictions on in-person research at this time, study participants were recruited by phone or email, which may have influenced their willingness to participate. The remote nature of the interviews, conducted by phone or videoconference, may have also impacted the sharing of information, as compared with an in-person interview. Furthermore, changes in care experienced due to social distancing and other restrictions may have increased interest in remote communications, including those that involved the collection of PGHD. Finally, we did not systematically collect participant clinical characteristics, such as pregnancy complications, that could impact participants’ perspectives about PGHD. Including the collection of these data in future studies may be important to understand the perspectives of individuals based on their specific clinical needs.

### Conclusions

Our qualitative analysis presents patient and provider perspectives on the collection of PGHD and its use during pregnancy. Participant responses collectively supported the use of PGHD to improve obstetric care. While several opportunities to expand the use of PGHD during pregnancy were mentioned, many considerations for implementing strategies to use PGHD were also noted, shedding light on the challenging nature of collecting and using this information in clinical practice.

## Appendix

Multimedia Appendix 1 Patient semistructured interview guide.

Multimedia Appendix 2 Provider semistructured interview guide.

Multimedia Appendix 3 Patient and provider coding dictionaries.

## Abbreviations

AMC: academic medical center
PGHD: patient-generated health data
