# Supplementary material for: Patient and Provider Perspectives About the Use of Patient-Generated Health Data During Pregnancy: Qualitative Exploratory Study
Source: JMIR Form Res. 2024 May 8;8:e52397. doi: 10.2196/52397 (PMC11112476; doi:10.2196/52397)
Supplement: Multimedia Appendix 1 [file formative_v8i1e52397_app1.docx]

**Patient Interview Guide**

**OVERVIEW OF INTERVIEW TOPICS**

In this interview, I will ask you a series of open-ended questions to get your perspectives about several topics. These topics include:

- Section 1: Information about you: Patient information
- Section 2: Experience with patient portals
- Section 3: Thoughts about collecting patient-reported outcomes

**PATIENT INTERVIEW QUESTIONS**

**Section 1: Information about you: Patient information**

- To start, I would like to ask for some demographic information:
  - How old are you?
  - What is your race?
  - Are you Hispanic or Latina?
  - What is your highest level or degree of education?
  - How far along are you in your pregnancy (or postpartum)?
  - Have you received care at the [clinic name] OB/GYN clinic?

**Section 2: Experience with patient portals**

*Patient portals are a tool that connects patients with their personal health information. You may have used a patient portal (e.g., MyChart) on your home computer or mobile device, or you may have used a patient portal in the hospital (e.g., MyChart Bedside) on a tablet computer or on a mobile device. These next questions are about your opinions of patient portals as a tool to engage in your health care during (or after) your pregnancy.*

- Have you ever used a patient portal?
  - If yes, which portal have you used? (e.g., OSU’s MyChart Bedside/MyChart or other)
  - If no, why not?
- How have you accessed a patient portal?
  - With internet access on a home computer?
  - With internet access on mobile device (e.g., phone/tablet)?
  - With internet access outside of your home (e.g., library/coffee shop)?
- In what settings have you used a patient portal? (e.g., at home or in the hospital)
- How have you used your patient portal to manage your health?
  - Making or checking appointment times?
  - Checking test results?
  - Reviewing medications or requesting medication refills?
  - Messaging your doctor?
  - Viewing education materials?

(For those who have not used a patient portal: How might a portal be useful to manage your health?)

- How did you learn to use the portal?
  - Did you receive any training? (If yes, please describe; If no, continue with questions below)
  - Would training be helpful?
  - What training would you need? (e.g., digital skills training to use internet, computer, or mobile devices; training to use patient portal features; training to understand patient portal features and how to use them to manage your health)
  - Who do you think you would you like to get training from?
- Are there features of your patient portal that are particularly helpful in the management of your health care during (or after) your pregnancy?
  - Are there particular health problems during your pregnancy that a patient portal could help you manage? (e.g., high blood pressure, diabetes, anxiety)
  - How could a patient portal help you attend your prenatal (or postpartum) care appointments?
  - How could a patient portal help with your self-knowledge of your health during (or after) your pregnancy?
- Are there additional features or functions of a patient portal that you think would be particularly helpful for patients during (or after) their pregnancy?
- Have you participated in video visits or telehealth calls since the beginning of the COVID-19 pandemic? (If yes, continue)
  - Were these visits conducted through the patient portal?
  - What did you think about these visits?

**Section 3: Thoughts about collecting patient-reported outcomes**

*Patient-reported outcomes are defined as any information regarding a patient’s health status that comes directly from the patient, without interpretation from the patient’s care provider. Providers sometimes request patient-reported outcomes from patients as a way to monitor their health and inform their care. For example, your doctor could send you a text message every day to ask you about your level of nausea. Your response about your level of nausea could help your doctor better manage your morning sickness. Another example might be the opportunity to report on fetal movement later in your pregnancy.*

*These next questions are about your opinions regarding patient-reported outcomes as a tool to engage patients in their health care during (or after) their pregnancy.*

- At your clinic visits, what are the types of information your providers regularly request during (or after) your pregnancy?
  - Do they ask about symptoms? Which ones? (e.g., nausea, depression, anxiety, pain, fatigue, dizziness/fainting, fever, bleeding, swelling of legs, hands, or face, headache, changes in vision, fetal movement)
  - Do they take measurements or do tests? What do they measure or test? (e.g., blood pressure or blood sugar)
- Has a care provider ever requested information from you between visits? For example, have they asked you to report your nausea, depression, anxiety, pain or other symptoms? Have they asked you to report blood pressure, blood sugar, or other measurements that you measured yourself?
  - Who talked with you about providing this information?
  - How was this information collected?
- What other type of information do you think would be helpful to report to your providers to help manage your health during (or after) your pregnancy? Is there anything they don’t ask you about that you wish they did? (e.g., have you ever had symptoms that you didn’t report to your provider, or they never asked you about?)
- Does/would reporting information about your health between visits make you feel more involved in your health care during (or after) your pregnancy?
  - If so, how?
- How would you like to report this information to your provider between visits?
  - Through a patient portal?
  - Text message?
  - Phone call?
  - Email?
- How often would you want to report this information to your provider?
  - Daily?
  - Weekly?

**INTERVIEW CLOSURE AND FOLLOW UP**

- Is there anything else you would like to share regarding the use of patient portals and patient-reported outcomes to enhance patient involvement in their health care during (or after) their pregnancy?
- Thank you for your time and participation! Your comments are extremely helpful to our study.
