# Supplementary material for: Patient and Provider Perspectives About the Use of Patient-Generated Health Data During Pregnancy: Qualitative Exploratory Study
Source: JMIR Form Res. 2024 May 8;8:e52397. doi: 10.2196/52397 (PMC11112476; doi:10.2196/52397)
Supplement: Multimedia Appendix 2 [file formative_v8i1e52397_app2.docx]

**Provider interview guide**

**OVERVIEW OF INTERVIEW TOPICS**

In this interview, I will ask you a series of open-ended questions to get your perspectives about several topics. These topics include:

- Section 1: Background
- Section 2: Thoughts about patient portals
- Section 3: Thoughts about patient-reported outcomes

**PROVIDER INTERVIEW QUESTIONS**

**Section 1: Background**

- To start, can you please tell me your role at OSUWMC in relation to treating obstetric patients?
  - How long have you been in that role?
- Do you provide care at the OSUWMC [clinic name] OB/GYN clinics?

**Section 2: Thoughts about patient portals**

*Your patients may use a patient portal to help manage their health care, either as an outpatient (e.g., MyChart) using their home computer or mobile device or as an inpatient (e.g., MyChart Bedside) through a tablet device. These next questions are about your experience with and opinions about patients using patient portals to help them engage in their health care.*

- Do you have a sense of how many of your patients use patient portals to manage their care?
- How do your patients use patient portals to manage their care?
  - Making or checking appointment times?
  - Checking test results?
  - Reviewing medications or requesting medication refills?
  - Messaging their doctor?
  - Viewing education materials?
- Do you think that patient portals help patients manage their care during or after their pregnancy?
  - If yes, how?
  - If no, why not?
- Are there other ways a patient portal would be particularly helpful for patients receiving care during or after their pregnancy?
  - Are there particular health problems during pregnancy that a patient portal could help patients manage? (e.g., high blood pressure, diabetes, anxiety)
  - How could a patient portal help patients attend their prenatal or postpartum care appointments?
  - How could a patient portal help with a patient’s self-knowledge of their health during or after their pregnancy?
  - How could a patient portal help with a patient’s anxiety during or after pregnancy?
- What training do providers get for using portals to assist patients receiving care at the OSUWMC [clinic name] OB/GYN clinic?
- What training do obstetric patients receiving care at the OSUWMC [clinic name] OB/GYN clinic get for using patient portals to manage their health? (e.g., digital skills training to use internet, computer, or mobile devices; training to use patient portal features; training to understand patient portal features and how to use them to manage your health)

**Section 3: Thoughts about collecting patient-reported outcomes**

*Patient-reported outcomes are defined as any information about a patient’s health status that comes directly from the patient, without interpretation from the patient’s care provider. Providers sometimes request patient-reported outcome data from patients as a way to monitor their health and inform their care. For example, a text message could be sent to a patient every day to ask about fetal movement. A patient’s response about their level of fetal movement could help alert doctors to potential complications in a patient’s pregnancy.*

*These next questions are about your experience with and opinions regarding patient-reported outcomes as a tool to engage patients in their health care during or after their pregnancy.*

- Do you collect patient-reported outcome data from your patients as a routine part of their care?
  - If yes, what patient-reported outcomes do you collect?
    - How do you collect them?
    - What do you do with them?
  - If not, would this be useful?
- Are there patient-reported outcomes that would be particularly helpful to collect from patients receiving care during or after their pregnancy?
  - Symptoms? (e.g., blood pressure, blood sugar, nausea, depression, anxiety, pain, fatigue, dizziness/fainting, fever, bleeding, swelling of legs, hands, or face, headache, changes in vision, fetal movement)
  - Other outcomes?
- Are there patient-reported outcomes that would be particularly helpful to collect between office visits for patients receiving care during or after their pregnancy?
- Would receiving patient-reported outcomes make you feel more involved in a patient’s care?
  - If so, how?
- How would you prefer to ask patients for their patient-reported outcomes?
  - Through a patient portal?
  - Text message?
  - Phone call?
  - Email?
- How could you use patient-reported outcomes in patient care?
- What types of challenges do you think might be encountered when collecting patient-reported outcome data?
- What types of challenges do you think might be encountered when using patient-reported outcome data in clinical practice?

**INTERVIEW CLOSURE AND FOLLOW UP**

- Is there anything else you would like to share regarding the use of patient portals and patient-reported outcome data to enhance patients’ involvement in their obstetric care?
- Thank you for your time and participation! Your comments are extremely helpful to our study.
